# A systematic review and meta-analysis of the diagnostic accuracy of pyruvate kinase M2 isoenzymatic assay in diagnosing colorectal cancer

**DOI:** 10.1186/s12957-015-0446-4

**Published:** 2015-02-13

**Authors:** Mallikarjuna Uppara, Franklin Adaba, Alan Askari, Susan Clark, George Hanna, Thanos Athanasiou, Omar Faiz

**Affiliations:** Surgical Epidemiology, Trials and Outcome Centre (SETOC), St Mark’s Hospital and Academic Institute, Harrow, Middlesex HA1 3UJ UK; Department of Surgery, St Mark’s Hospital and Academic Institute, Harrow, Middlesex HA1 3UJ UK; Department of Surgical Sciences, Imperial College, St Mary’s Hospital, Praed Street, London, W21NY UK; Department of Cardiovascular Sciences and Cardiac Surgery, Imperial College, St Mary’s Hospital, Praed Street, London, W21NY UK; North West London Hospitals Trust, Harrow, Middlesex HA1 3UJ UK

**Keywords:** Colorectal cancer, Faecal test, Pyruvate kinase isoenzymatic assay

## Abstract

**Background:**

Screening programmes exist in many countries for colorectal cancer. In recent years, there has been a drive for a non-invasive screening marker of higher sensitivity and specificity. Stool-based pyruvate kinase isoenzyme M2 (M2-PK) is one such biomarker under investigation. The aim of this systematic review and meta-analysis is to determine the diagnostic accuracy, sensitivity and specificity of M2-PK as a screening tool in colorectal cancer.

**Methods:**

A literature search of Ovid Medline, EMBASE and Google Scholar was carried out. The search strategy was restricted to human subjects and studies published in English. Data on sensitivity and specificity were extracted and pooled. Statistical analysis was conducted using summary receiver operating characteristic (SROC) curve methodology.

**Results:**

A total of eight studies were suitable for data synthesis and analysis. Our analysis showed a pooled sensitivity and specificity for M2-PK to be 79% (CI 73%–83%) and 80% (CI 73%–86%), respectively. The accuracy of M2-PK was 0.85(0.82–0.88).

**Conclusion:**

Faecal M2-PK assay has a relatively good sensitivity and specificity and high accuracy for screening colorectal cancer.

## Review

### Introduction

Worldwide, colorectal cancer (CRC) is a common global cancer accounting for approximately 694,000 deaths annually (World Health Organisation). Early detection of cancer is important for cancer survival; as a result, screening programmes have been set up in a variety of countries around the world. Whilst colonoscopy has been used as a screening tool in some countries, it is invasive, has inherent (although low) risks of perforation [1] and the uptake by the population is low [2].

Another form of population screening is faecal occult blood testing (FOBT) using either guaiac-based or immunological-based (iFOBT) methods. Guaiac-based faecal occult blood (FOBT) has been shown to have a sensitivity of 7.2%, whilst the newer iFOBT has a sensitivity of 23.2% for colorectal cancer and significant precursor lesions [3]. According to a meta-analysis [4], the sensitivity, specificity and positive predictive value of immune chromatographic faecal occult blood test (iFOBT) in CRC were 67%, 85% and 41%, respectively. The low sensitivity of FOBT and iFOBT may result in missing patients with colorectal cancers. Therefore, an effective screening tool is necessary.

In recent years, efforts have been made to introduce a more sensitive and specific marker in CRC screening. One marker that has been investigated is pyruvate kinase (M2-PK), an isoenzyme found in tumour cells. M2-PK is a key enzyme within glycolysis, a process that catalyzes the conversion of phosphoenolpyruvate (PEP) to pyruvate. Depending upon the metabolic functions of the tissues, different isoenzymes of pyruvate kinase are expressed. During tumour formation, the tissue-specific isoenzymes disappear and the pyruvate kinase isoenzyme type M2 is expressed [5]. M2-PK is crucial for rapid tumour growth and aerobic glycolysis during tumorigenesis [6,7].

Tumour M2-PK has been detected and quantified in faeces (5). This has led to the development of faecal M2-PK as a screening tool for colorectal cancer using enzyme-linked immunoabsorbent assay (ELISA). However, there appears to be some contention in the literature regarding its utility. Some studies on faecal M2-PK have shown a favorable sensitivity and specificity for colorectal cancer and adenoma [8,9] whilst other studies have shown an unfavorable sensitivity and specificity of faecal M2-PK [10-12].

The aim of this systematic review and meta-analysis is to determine the overall sensitivity and specificity of M2-PK in colorectal cancer and its potential as a population screening tool.

### Methods

#### Search strategy

A comprehensive literature search of PubMed, MEDLINE (1946-Dec 2012), EMBASE (1974-Dec 2012) and Google Scholar was carried out for articles in the English language. The terms and keywords used included Pyruvate Kinase, M2-PK, colorectal cancer, colon cancer, rectal cancer and pyruvate kinase M2.

#### Selection criteria and quality assessment

Articles with sensitivity and specificity results of faecal M2-PK for colorectal cancer with confirmation of cancer using colonoscopy and histology were included in our study. Studies on plasma M2-PK, adenoma only, functional bowel disorders only and inflammatory bowel diseases only were excluded along with posters, systematic reviews, meta-analysis, articles not in English and other gastrointestinal cancers. Quality assessment of the included studies was carried out using ‘QUADAS’ scoring system (Table 1).

**Table 1:** **Quality assessment of included studies using ‘QUADAS’ questionnaire**

**QUADAS questionnaire**	**Abdullah et al. 2012 [** 13 **]**	**Parente et al. 2012 [** 14 **]**	**Mulder et al. 2007 [** 10 **]**	**Hardt et al. 2004 [** 15 **]**	**Koss et al. 2007 [** 16 **]**	**Tonus et al. 2006 [** 17 **]**	**Shastri et al. 2008 [** 11 **]**	**Haug et al. 2007 [** 18 **]**
Was the spectrum of participants representative of the patients who will receive the test in practice?	Yes	Yes	Yes	Yes	Yes	Yes	Yes	Yes
Were selection criteria clearly described?	Yes	Yes	Yes	No	Yes	Yes	Yes	Yes
Was the reference standard likely to classify the target condition correctly?	Yes	Yes	Yes	Yes	Yes	Yes	Yes	Yes
Was the period between performance of the reference standard and the index test short enough to be reasonably sure that the target condition did not change between the two tests?	Yes	Yes	Yes	Yes	Yes	Yes	Yes	Yes
Did the whole sample or a random selection of the sample receive verification using the reference standard?	Yes	Yes	Yes	Yes	Yes	Yes	Yes	Yes
Did participants receive the same reference standard regardless of the index test result?	Yes	Yes	Yes	Yes	Yes	Yes	Yes	Yes
Was the reference standard independent of the index test? (That is, the index test did not form part of the reference standard)	No	Yes	Yes	Yes	Yes	No	Yes	Yes
Was the execution of the index test described in sufficient detail to permit its replication?	Yes	Yes	Yes	Yes	Yes	Yes	Yes	Yes
Was the execution of the reference standard described in sufficient detail to permit its replication?	Yes	Yes	Yes	Yes	Yes	Yes	Yes	Yes
Were the index test results interpreted without knowledge of the results of the reference standard?	No	Unclear	Yes	No	No	No	No	No
Was the reference standard results interpreted without knowledge of the results of the index test?	No	No	No	No	No	Unclear	Yes	Unclear
Were the same clinical data available when the test results were interpreted as would be available when the test is used in practice?	Yes	Yes	Yes	Yes	Yes	Yes	Yes	Yes
Were un-interpretable, indeterminate or intermediate test results reported?	No	Yes	Yes	No	No	No	Yes	Unclear
Were withdrawals from the study explained?	Unclear	Yes	Yes	No	No	No	Yes	Unclear

#### Data extraction and analysis

Data were extracted and reviewed by two reviewers (MU and FA), and disagreements were resolved through discussion and consensus between the authors. Studies that did not contain relevant data or where data were not amenable to extraction were excluded. A pooled result of sensitivity, specificity and diagnostic accuracy of M2-PK from all included studies was done using Stata 13.1 statistical software. Results of the post-estimate are depicted as forest plots, SROC curve and foreb plots. A *p* value of <0.05 was deemed statistically significant.

#### Data synthesis

**Figure 1:**
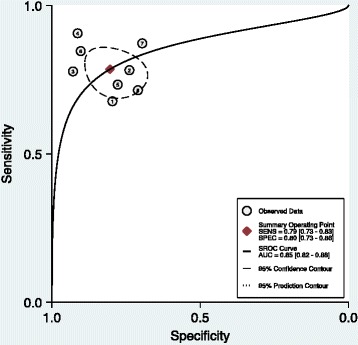
**SROC curve of M2-PK isoenzymatic assay of faeces samples for following eight studies: Haug et al. [** 18 **], Shastri et al. [** 11 **], Tonus et al. [** 17 **], Koss et al. [** 16 **], Hardt et al. [** 15 **], Mulder et al. [** 10 **], Parente et al. [** 14 **] and Abdullah et al. [** 13 **].**

A total of eight studies were suitable for data synthesis. Pooling of sensitivity and specificity results from the eight studies showed a combined sensitivity of M2-PK as 79% (CI 73%–83%) and the combined specificity to be 80% (CI 73%–86%) for M2-PK. The accuracy was 0.85 (0.82–0.88) (Figure 1).

### Results

**Figure 2:**
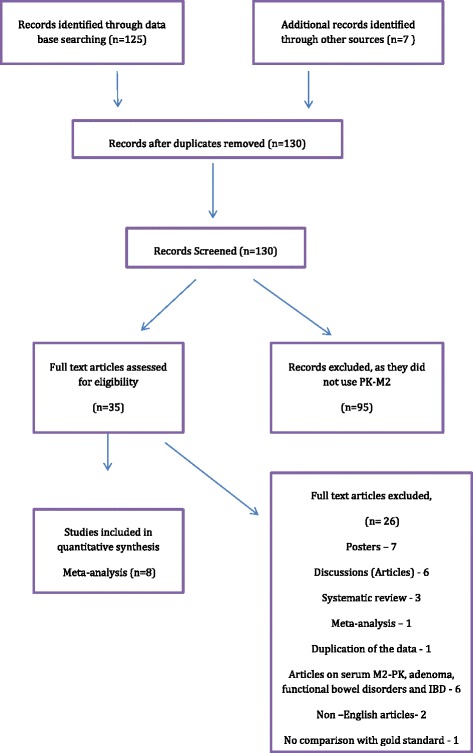
**A PRISMA diagram outlining the search strategy.**

Search results returned a total of 132 articles, of which 125 were obtained through electronic searches and seven were from manual reference matching (Figure 2). After removal of duplicates, 130 abstracts were screened. Of these, 35 articles were eligible for review of full text with eight studies suitable for meta-analysis.

#### Demographics

A total of 2,654 patient data were pooled from the eight articles. Of these, 407 patients had a confirmed diagnosis of colorectal cancer on colonoscopy and histology. Using M2-PK enzymatic assay, 317 (11.94%) of participants were identified as true positives, 526 (19.82%) as false positives, 1,721 (64.85%) as true negatives and 90 (3.39%) as false negatives.

#### Sensitivity

**Figure 3:**
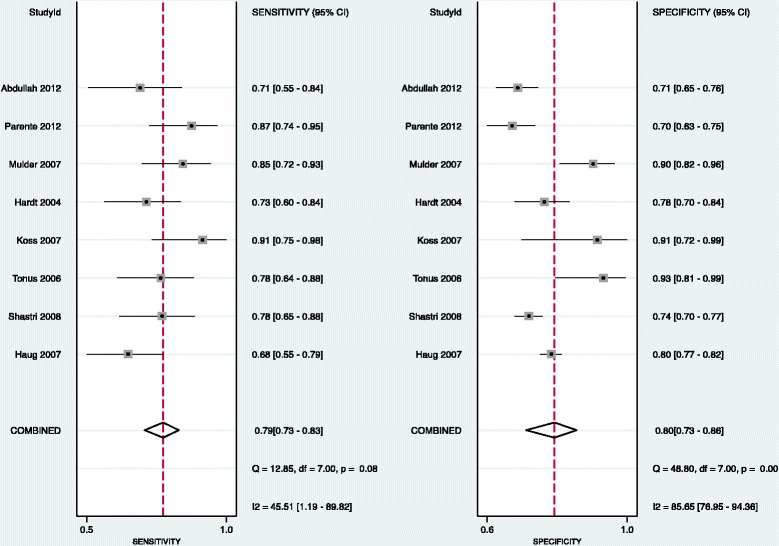
**Forest plots showing the sensitivity and specificity for individual studies with their 95% confidence intervals, combined sensitivity and combined specificity from all the included studies.**

Pooling of data demonstrated the combined sensitivity of M2-PK as a screening to be 79% (CI 73%–83%), *Q* = 12.85, *df* = 7.00, *p* = 0.08 (Figure 3) with *I*^2^ = 45.51 (1.19–89.82). There was moderate heterogeneity in the included studies. We also assessed the empirical Bayes of the sensitivity of the included studies and compared it with that of observed data (Figure 3) and found that the observed sensitivity was overestimated in three studies (study number 4, 6 and 7).

#### Specificity

**Figure 4:**
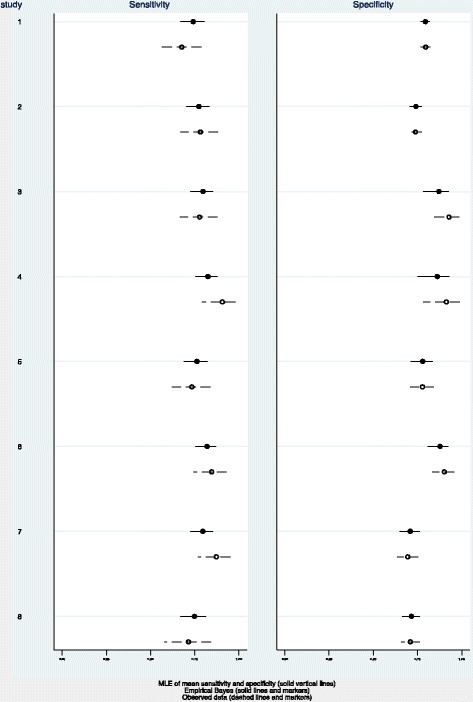
**Foreb plot.**

**Figure 5:**
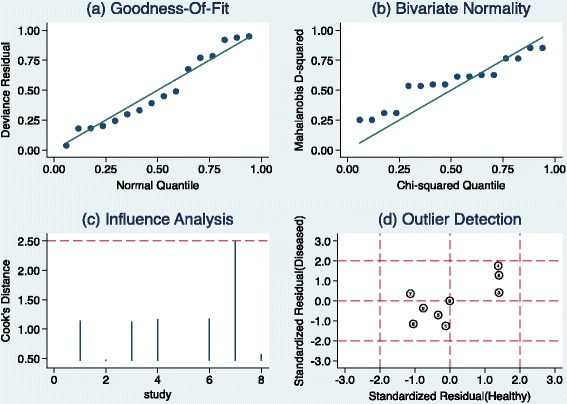
**Graphical depictions of residual-based goodness-of-fit, bivariate normality, influence and outlier detection analyses.**

Pooling of data also demonstrated a combined specificity of 80% (CI 73%–86%), *Q* = 48.80, *df* = 7.00, *p* = <0.001 (Figure 3) with *I*^2^ = 85.65 (76.95–94.36). There was considerable heterogeneity in the included studies. We also assessed the empirical Bayes of the specificity of the included studies and compared them with that of observed data (Figure 4). The observed specificity was overestimated in three studies (studies 3, 4 and 6). These three studies were found to be outliers (Figure 5).

#### Accuracy

The diagnostic accuracy was calculated by measuring the area under the curve (Figure 1) from the included studies. The diagnostic accuracy of M2-PK for diagnosing colorectal cancer was 0.85 (0.82–0.88).

#### Subgroup analysis

Removal of outliers from Figure 1 (studies numbered 3, 4, 6 and 7) has removed the effect of outliers outside the 95% prediction contour on the ROC curve in measuring overall accuracy. All the studies were lying within the 95% prediction contour of measured accuracy (adjusted accuracy), though it is lower than the observed accuracy for eight studies.

**Figure 6:**
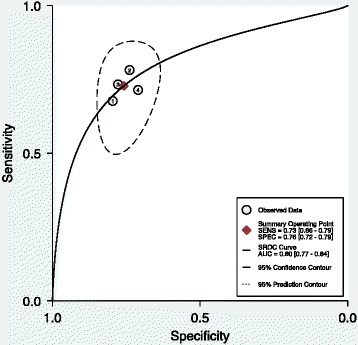
**SROC curve of M2-PK isoenzymatic assay of faeces samples for the following four studies (excluding the outliers): Haug et al. [** 18 **], Shastri et al. [** 11 **], Hardt et al. [** 15 **] and Abdullah et al. [** 13 **].**

**Figure 7:**
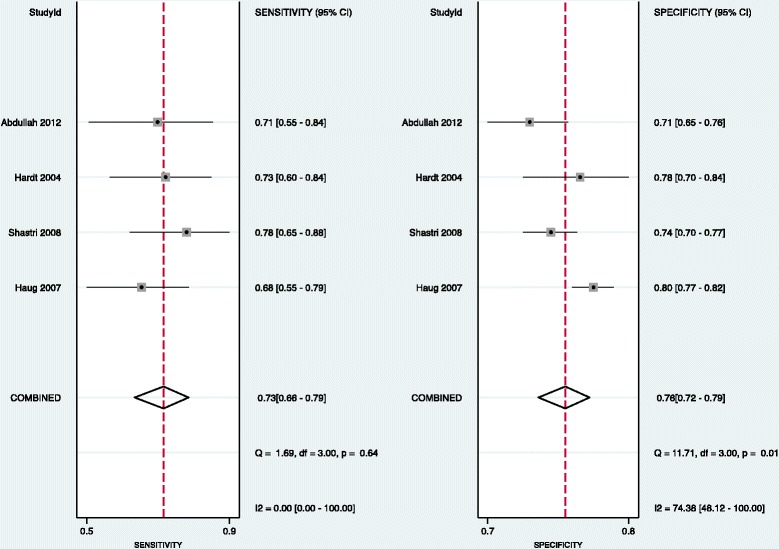
**Forest plots showing the sensitivity and specificity for individual studies with their 95% confidence intervals, combined sensitivity and combined specificity from four studies excluding the out liars.**

The adjusted accuracy (Figure 6) was 0.80 (0.77–0.84). The adjusted sensitivity was 0.73 (0.66–0.79) (Figure 7), and the adjusted specificity was 0.76 (0.72–0.79) (Figure 7).

Heterogeneity whilst estimating sensitivity was nullified after the removal of the outliers, but significant heterogeneity remained whilst estimating specificity (although *p* value is 0.01).

### Discussion

The result of this study suggests that M2-PK may have a high sensitivity and specificity (significant heterogeneity was noted whilst estimating specificity) for diagnosing colon cancer. Current screening methods such as FOBT and iFOBT have a lower documented sensitivity, specificity rates as well as a lower positive predictive value [19]. Bleeding from the bowel due to non-malignant causes can give rise to false positives in FOBT and iFOBT. Conversely, small tumours may not cause significant bleeding due to the absence of tumour necrosis or angiogenesis and therefore result in a false-negative test on FOBT. Tumour necrosis and angiogenesis are late events in the tumour progression, which lowers the sensitivity of FOBTs for non-bleeding tumours. In many countries such as the UK, positive FOBT is a prerequisite to further invasive investigations such as colonoscopy. High false-positive rates, therefore, result in potentially unnecessary colonoscopies, which may be unpleasant for the patient and are also costly.

Therefore, a screening modality that is non-invasive, easy to use and possesses a high sensitivity and specificity rate as well as a high predictive value is desirable. M2-PK has been identified in stool samples of patients with colorectal cancer with a strong correlation between the amount of faecal tumour M2-PK and tumour stage [15]. Our results show M2-PK faecal assays to have a sensitivity and specificity of 79% and 80%, respectively, at a cutoff value of 4 units/μl, giving a higher sensitivity than FOBT but a lower specificity in comparison. This value is in keeping with studies such as Tonus et al. [11,12,15,17]. With these sensitivity and specificity values, M2-PK may therefore be an effective addition to screening for bowel cancer along with FOBT and iFOBT so that both sensitivity and specificity of the screening tools can be kept at an optimum level as required.

Furthermore, FOBT has been associated with a particularly low cancer pick-up rate in proximal cancers [3]. This appears to be a lesser problem with M2-PK as it has a high sensitivity rate for proximal tumours [18,10]. In combination with iFOBT, PK-M2 may enhance the overall pickup rate for proximal cancers [14].

However, there are cost implications associated with M2-PK for use as a screening tool. A study by Koss and colleagues in 2007 [16] showed the cost of M2-PK test to be £13.50 per sample. This is substantially higher than the cost of FOBT (£5.00 per person) [16]. But costs of the colonoscopy or flexible sigmoidoscopy may outweigh the costs of M2-PK if applied for false-positive patients with FOBT or iFOBT and, if avoided, an unnecessary colonoscopy. Colonoscopy and flexible sigmoidoscopy is more expensive than either FOBTs or M2-PK due to the requirement of both technical expertise and instrumentation costs. Also there is a risk of bowel damage/perforation with invasive procedures such as colonoscopy and flexible sigmoidoscopy.

Diagnostic accuracy is a good estimate of the true nature of the test, though it does take into the account the amount of heterogeneity whilst estimating combined accuracy. M2-PK accuracy for diagnosing colorectal cancer is 0.85. In this meta-analysis, we have analysed colorectal cancer data from the included papers separately from the data for polyps and other benign conditions such as inflammatory bowel disease. Amongst the included papers, some papers have also reported that faecal M2PK levels are slightly elevated in benign conditions as well, although their levels are not as high as that of colorectal cancer.

## Conclusion

In conclusion, faecal M2-PK testing has a high sensitivity and specificity rate as well as a high accuracy for diagnosing bowel cancer in a screened population. Although more costly than FOBT, it has the potential to be used as a screening tool in the general population.

### Limitations of the study

1. Selection bias could have led to the recruitment of more symptomatic patients in UK-based studies. Henceforth, studies done in UK have reported very high sensitivity and high specificity when compared to other studies.
2. A lack of randomisation is another major drawback in these studies if one needs to compare strictly against gold standard (colonoscopy) whilst assessing the accuracy of the test.
3. Significant false positives were also reported in some of the studies with the generic test ‘M2PK’ in non-neoplastic conditions such as inflammatory bowel diseases.
4. Cutoff values for ‘M2PK’ were not standardised to differentiate between neoplastic and inflammatory conditions of the bowel.
5. Estimation of ‘M2PK’ enzymatic activity by ELISA technique (which was used in all the included studies) has a disadvantage of reporting high false positives as opposed to more accurate methods such as PCR techniques.
